# Takotsubo Syndrome Presenting as ST‐Elevation Myocardial Infarction With Concomitant Persistent High‐Grade AV Block Requiring Cardiac Resynchronisation Therapy Pacemaker

**DOI:** 10.1155/cric/1482184

**Published:** 2026-02-27

**Authors:** Jhiamluka Solano, Ali Hussain, Adnan Ahmed, Raj Chelliah, Muhammad Usman Shah

**Affiliations:** ^1^ Radcliffe Department of Medicine, Oxford Centre for Clinical Magnetic Resonance Research, University of Oxford, Oxford, UK, ox.ac.uk; ^2^ Hull University Teaching Hospitals NHS Trust, Hull, UK; ^3^ Lincoln Heart Center, University of Lincoln, Lincolnshire, UK, lincoln.ac.uk

## Abstract

Takotsubo syndrome (TTS), also known as stress‐induced cardiomyopathy or apical ballooning syndrome, is increasingly recognised as an acute myocardial ischemic syndrome primarily involving transient microvascular dysfunction rather than epicardial coronary occlusion and characterised by transient left ventricular dysfunction. Patients are predominantly postmenopausal women and may clinically mimic acute coronary syndrome, including ST‐elevation myocardial infarction (STEMI) as well as non–ST‐elevation myocardial infarction (NSTEMI). While TTS may coexist with obstructive coronary artery disease (CAD), the hallmark feature is a mismatch between the severity of wall motion abnormalities and the distribution of any coronary lesions. We present a postmenopausal lady who presented as a presumed STEMI but was eventually diagnosed to have takotsubo cardiomyopathy. She developed a persistent high‐grade AV block with poor ejection fraction and was subsequently implanted with a cardiac resynchronisation therapy pacemaker (CRT‐P).

## 1. Introduction

Takotsubo syndrome (TTS) is a distinct form of acute heart failure syndrome characterised by transient regional systolic dysfunction of the left ventricle (LV) [1]. Usually, it occurs secondary to severe emotional or physical stress. The presence of bradyarrhythmias, especially high‐degree atrioventricular block, is uncommon in patients with TTS [2]. The presence of atrioventricular block with TTS may pose a therapeutic dilemma with respect to the need for a pacemaker. We present a case of TTS with persistent high‐grade AV block presented as an acute coronary syndrome and treated with device therapy.

## 2. Case Presentation

A middle‐aged postmenopausal lady presented to the emergency department of a local district general hospital feeling generally unwell, with light‐headedness and sweating. She had been having progressive symptoms over the past 2–3 months and was under follow‐up at the local general practitioner (GP) surgery. She denied any history of chest pain, shortness of breath, syncope or seizures. There was no history of nausea, vomiting, vertigo, otalgia, otorrhea and tinnitus. Dizziness was not associated with a change in posture, and there was no history of viral illness recently.

**Figure 1:**
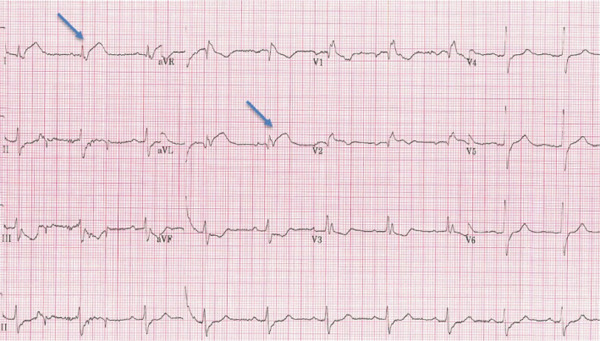
Twelve‐lead ECG showing ST elevation (blue arrows) in Leads 1 and aVL.

Her baseline vitals on arrival included a temperature of 37°C, blood pressure of 130/80 mmHg, heart rate of 70 beats/minute and respiratory rate of 18 breaths/minute. On clinical examination, the patient looked anxious and tired. Her general physical examination and HEENT (head, eyes, ear, nose and throat) examination were normal. Heart tones were normal, and no added sounds or murmurs were appreciated. Her central nervous system examination was unremarkable for nystagmus or any focal neurological deficit. She was known to have essential hypertension on losartan only and osteoarthritis and was a nonsmoker with no alcohol intake. Her 12‐lead ECG (Figure 1) showed a RBBB, prolonged PR interval and new ST elevation in lateral Leads I and avL with reciprocal ST depression in inferior Leads II, III and avF. She was loaded with aspirin 300 mg and ticagrelor 180 mg orally and accepted immediately for transfer to the local tertiary care centre for emergent angiography.

**Figure 2:**
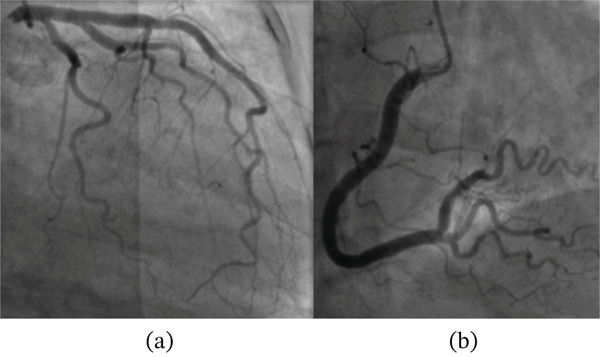
Invasive coronary angiography with caudal view of left coronary arteries (a) and cranial view of right coronary artery (b) showing no significant stenosis.

**Figure 3:**
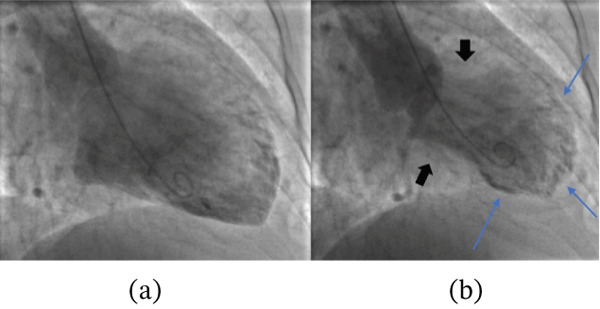
Left ventriculography in diastole (a) and systole (b) with good contraction of basal segments (black arrows) and poor contraction of mid and apical segments (blue arrows) of the left ventricle with resulting apical ballooning, typical of TTS.

**Figure 4:**
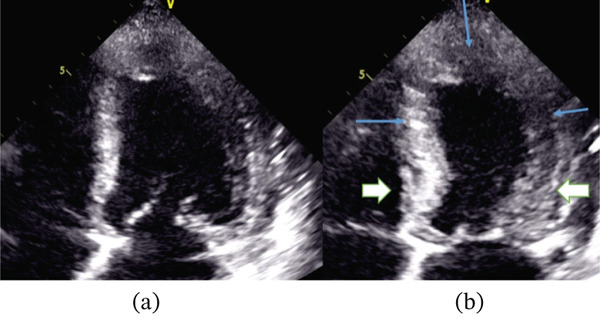
Transthoracic echocardiography showing apical four‐chamber view of the left ventricle in diastole (a) and systole (b) showing good basal contractions (black arrows) but poor mid and apical segment contractions (blue arrows).

**Figure 5:**
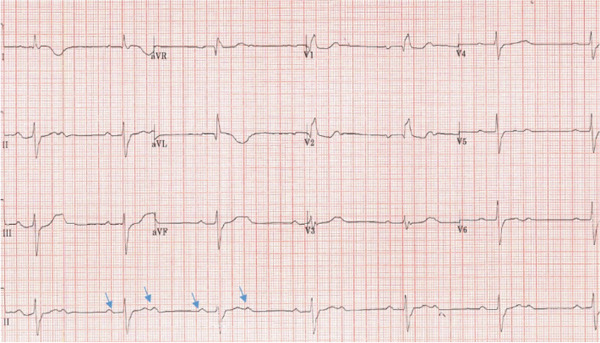
Twelve‐lead ECG showing 2:1 Mobitz Type 2 second degree heart block (blue arrows represent P waves).

Her angiogram showed normal coronary arteries with no luminal obstruction (Figure 2). Therefore, a left ventriculogram was done (Figures 3 and 4), which showed hypokinesia of anterior, apical and apical cap associated with moderate to severe left ventricular systolic dysfunction (LVSD) with similar findings on transthoracic echocardiography. Her troponin was reported elevated at 147 ng/L (limit of detection 5–14 ng/L, 99th centile value), which was disproportionately small relative to the extent of left ventricular dysfunction, and the rest of the blood tests were all normal. Subsequent 12‐lead ECGs captured Mobitz Type 2 second‐degree heart block (Figure 5) with persistent RBBB and prolonged QRS of 162 ms.

The crucial diagnostic challenge is distinguishing TTS from STEMI, myocardial infarction with nonobstructive coronary arteries (MINOCAs) and myocarditis. Typical ECG changes, as in our case with elevated troponin levels, may be present in such conditions. However, TTS and MINOCA have normal or nonobstructive coronary arteries on angiography, unlike myocardial infarction, which typically shows disruptive atherosclerotic plaque and flow limitation secondary to ruptured plaque or thrombus. Furthermore, invasive left ventriculography revealed regional wall motion hypokinesia with the typical apical ballooning characteristic of TTS. In contrast, STEMI shows regional wall motion abnormalities without apical ballooning, and myocarditis shows global changes.

Post angiography, she was shifted to the cardiac coronary unit (CCU), observed on telemetry and started on heart failure treatment. Her history was revisited for any precipitating factor (recent emotional stress or possible triggers); however, she denied any such events. It is worth noting that the patient had an InterTAK Diagnostic Score of 25, corresponding to an intermediate pretest probability of TTS.

During observation, the patient developed recurrent dizziness, and telemetry demonstrated a 2:1 high‐grade second‐degree atrioventricular block (Figure 5). The conduction abnormality persisted despite atropine administration, consistent with a nonnodal (infra‐Hisian) level of block, and despite withdrawal of recently initiated beta‐blocker therapy commenced as part of guideline‐directed medical therapy (GDMT) for heart failure with reduced ejection fraction.

The patient was discussed in a local electrophysiology multidisciplinary team meeting (EP‐MDT), and it was decided to implant a cardiac resynchronisation therapy pacemaker (CRT‐P) in the context of moderate to severely reduced left ventricular systolic function, RBBB morphology with prolonged QRS duration of 162 ms and symptomatic persistent high‐grade AV block requiring cardiac pacing based on the European Society of Cardiology guidelines. After successful device implantation, she was discharged home on GDMT for heart failure with reduced ejection fraction, including bisoprolol 2.5 mg once daily, bumetanide 1 mg once daily, enalapril 2.5 mg twice daily (changed from regular losartan) and eplerenone 25 mg once daily.

She was followed up in the device clinic after 6 weeks with no arrhythmia detected and remained clinically stable. Her repeat echocardiogram showed an improved ejection fraction of 50% and improvement in the mid‐to‐apical hypokinetic segments. She remained asymptomatic, and the pacemaker check was satisfactory. An outpatient cardiac MRI was performed (MRI‐conditional CRT‐P system), which showed borderline impaired function with ejection fraction of 53% and no scar or necrosis on late gadolinium enhancement, hence confirmatory for TTS. She was scheduled for further follow‐up in the device and heart failure clinics subsequently. At her follow‐up appointment, she had not had a new occurrence of syncope, AVB or TTS. Furthermore, her pacing checks on the year of implant revealed a right atrium pacing percentage of 96%, right ventricle pacing percentage of 100% and a coronary sinus pacing percentage of 100%. Four years later, this value minimally changed to 99% across all leads.

The patient provided her perspective of care. She said: “I am happy with the treatment I received and the care on the ward. Subsequent visits to the pacing clinic have been helpful, and various difficulties have been tweaked. However, I am certainly more tired than I was, have less energy and walk fewer miles than before all this happened. It is difficult to know if this is just because I am getting older or if it is connected in any way.”

## 3. Discussion

The term tako tsubo is the Japanese name for a pot used to trap octopuses, whose silhouette resembles the typical apical ballooning observed in these patients at ventriculography. TTS is a well‐described clinical syndrome and accounts for 1%–2% of patients presenting with features suggestive of myocardial infarction [1].

According to the International Takotsubo Registry study (a consortium of 26 centres in Europe and the United States), the most common symptoms among patients diagnosed with TTS were chest pain, dyspnoea and syncope (75.9%, 46.9% and 7.7%, respectively) [2].

On initial presentation, few patients develop symptoms and signs consistent with heart failure, and cardiogenic shock is present in approximately 10% of patients. Furthermore, subsets of patients develop serious cardiac arrhythmias, which include both tachyarrhythmias (including ventricular tachycardia and ventricular fibrillation) and bradyarrhythmias (advanced heart block) and even sudden cardiac arrest [3, 4].

Association of TTS with advanced AV heart block is uncommon; however, several case reports since 2009 have highlighted this rare but clinically relevant association [5, 6]. Atrioventricular block is reported in 2.9% of the total cases of TTS, and its association with the latter is elusive [7]. The exact etiopathogenesis is complex and rather difficult to explain as TTS is believed to be caused by enhanced sympathetic tone and involves the mid and apical aspects of the LV, which is anatomically far away from the AV node. Another plausible mechanism of AV conduction disturbance could be diffuse spasm in small coronary branches, causing ischemia and an increase in vagal tone [8].

It is important to note that bradyarrhythmia can resolve on its own; however, fatal bradyarrhythmia can reoccur and cause sudden cardiac death [9]. Furthermore, the occurrence of life‐threatening arrhythmias depends on several factors, including lower ejection fraction, longer QTc interval, PR interval and longer R‐R interval [10].

Our patient had an LVEF of 30%–35% with RBBB and a prolonged QRS duration, warranting CRT‐P implantation. The persistence of near‐complete ventricular pacing over long‐term follow‐up strongly suggests underlying intrinsic conduction‐system disease, but the temporal association with TTS does not establish causation. While causality cannot be inferred from a single case, pacemaker interrogation showed persistent high biventricular pacing (RV and CS 100% at 1 year; 99% at 4 years), indicating ongoing complete AV block despite clinical stability. Echocardiography showed LVEF recovery to 50%, corroborated by cardiac MRI (EF 53%), suggesting that LV dysfunction resolved independently of AV conduction recovery.

Moreover, in most cases, AV block persists once the left ventricular function is normalised, leading to permanent pacemaker implantation and indicating that recovery of left ventricular function is not parallel with the resolution of the AV conduction disturbances. For example, in a case report of TTS with AV block, a temporary pacemaker was inserted during an early recovery period and eventually changed to a permanent pacemaker after 18 days because of persistent AV block. Furthermore, an electrophysiological study conducted by Nault et al. demonstrated a lack of resolution of a high‐degree AV block 1 year after TTS, which was eventually resolved 2 years later [11]. It is highly likely that the recovery of the cardiac conduction system is prolonged and lags that of the myocardial tissue recovery in patients with TTS. Therefore, decisions should be made on a case‐by‐case basis based on clinical judgement and lack of conduction improvement in the electrophysiological study [12]. It is noteworthy that as there is a strong link between catecholamine excess and TTS, beta‐blockers are advocated as the mainstay of treatment.

Recent data suggest that advanced atrioventricular block is not associated with worse hospital outcomes, unlike what occurs with ventricular arrhythmias [13]. Additionally, while the InterTAK Diagnostic Score [14] yielded an intermediate probability of TTS, predominantly driven by female sex, the diagnosis was established through integration of angiographic, ventriculographic, biomarker and cardiac MRI findings rather than reliance on the score alone. Importantly, the decision to implant a CRT‐P was based on established pacing principles in the setting of persistent high‐grade atrioventricular block, broad right bundle branch block (QRS 162 ms), reduced left ventricular ejection fraction and an anticipated near‐complete ventricular pacing burden [15, 16]. In this context, CRT‐P was favoured over conventional right ventricular pacing to mitigate the risk of pacing‐induced cardiomyopathy, with subsequent recovery of left ventricular systolic function supporting physiologically appropriate ventricular activation despite persistent conduction disease.

TTS is not a common disease, and its association with the high‐degree atrioventricular block might be a rarer phenomenon. Overall, the prognosis of TTS is good; however, extra care should be taken when coexistent conductive tissue disease occurs as the need for permanent pacemakers may arise, and the fatality rate may also be high due to malignant arrhythmias. Evidence on the management of TTS and conductive tissue abnormalities with a permanent pacemaker is limited to case reports. Therefore, in the absence of established guidelines, more research is needed to formalise standardised management strategies in such patients.

## 4. Learning Points

• TTS is an uncommon condition, and its association with high‐degree atrioventricular block is even rarer.
• The prognosis of TTS is good; however, extra care should be taken when there is coexistent conductive tissue disease, as it has an impact on morbidity and mortality.
• Evidence on the management of TTS and cardiac conduction abnormalities with a permanent pacemaker is limited to case reports.
• In cases of persistent significant heart block, prolonged QRS duration and left ventricular dysfunction, a CRT may be preferred over a dual‐chamber pacemaker.

## Author Contributions

Jhiamluka Solano and Muhammad Usman Shah were responsible for the conception of the case report, literature review and drafting of the manuscript. Ali Hussain, Adnan Ahmed, Raj Chelliah and Muhammad Usman Shah contributed to patient management, data acquisition and critical revision of the manuscript for important intellectual content.

## Funding

No funding was received for this work.

## Disclosure

All authors reviewed and approved the final version of the manuscript and agree to be accountable for all aspects of the work.

## Consent

All the patients allowed personal data processing, and informed consent was obtained from all individual participants included in the study.

## Conflicts of Interest

The authors declare no conflicts of interest.
